# Phenols and Polyphenols as Carbonic Anhydrase Inhibitors

**DOI:** 10.3390/molecules21121649

**Published:** 2016-12-02

**Authors:** Anastasia Karioti, Fabrizio Carta, Claudiu T. Supuran

**Affiliations:** 1 Laboratory of Pharmacognosy, School of Pharmacy, Aristotle University of Thessaloniki, University Campus, Thessaloniki 54124, Greece; 2 Neurofarba Department, Sezione di Chimica Farmaceutica e Nutraceutica, Università degli Studi di Firenze, Via U. Schiff 6, I-50019 Sesto Fiorentino (Firenze), Italy; fabrizio.carta@unifi.it

**Keywords:** carbonic anhydrase inhibitors (CAIs), metalloenzymes, phenols, polyphenols, flavonoids

## Abstract

Phenols are among the largest and most widely distributed groups of secondary metabolites within the plant kingdom. They are implicated in multiple and essential physiological functions. In humans they play an important role as microconstituents of the daily diet, their consumption being considered healthy. The physical and chemical properties of phenolic compounds make these molecules versatile ligands, capable of interacting with a wide range of targets, such as the Carbonic Anhydrases (CAs, EC 4.2.1.1). CAs reversibly catalyze the fundamental reaction of CO_2_ hydration to bicarbonate and protons in all living organisms, being actively involved in the regulation of a plethora of patho/physiological processes. This review will discuss the most recent advances in the search of naturally occurring phenols and their synthetic derivatives that inhibit the CAs and their mechanisms of action at molecular level. Plant extracts or mixtures are not considered in the present review.

## 1. Introduction

### 1.1. The Carbonic Anhydrases Family, Occurrence and Role

CO_2_, bicarbonate and protons are an essential molecule and ions for many important physiologic processes occurring in all living organisms, including *Archaea*, *Bacteria*, and *Eukarya* [1,2]. However the uncatalyzed rate of interconversion of such species is too slow to meet the physiological needs of most biochemical processes [2,3]. This task is efficiently accomplished by the carbonic anhydrases (CAs, EC 4.2.1.1). This superfamily of metalloenzymes possess within their active sites a highly nucleophilic metal hydroxide species, such as zinc(II), cadmium(II) or iron (II) hydroxide, depending on the class [2,3]. To date seven not-genetically related CA families are reported, indicated with the Greek letters α-, β-, γ-, δ-, ζ- η- and θ- [1,2,3,4,5,6,7,8,9]. All the CA families have been thoroughly characterized by means of kinetic and structural features, with the exception of the δ-, η- and θ-CAs, for which only kinetic data are available so far [8,9,10,11]. The α-CAs are mainly distributed among the vertebrates, protozoa, algae, cytoplasm of green plants and in many *Bacteria* species. Since α-CAs are the only superfamily expressed in humans, they are also the most investigated ones for medicinal chemistry purposes [1,2,3,4,5,8,12,13,14,15,16,17]. Mammals (except the primates) possess 16 α-CA-isoforms, and among them three are devoid of catalytic activity [1,2]. The distribution of the various isoforms differs within tissues, cellular compartments as well as in modification of physio/pathological conditions [1,2]. The inhibition and activation mechanisms of CAs are well understood processes at a molecular level. The classical CA inhibitors (CAIs) such as the primary sulfonamides and their bioisosters (the sulfamides and sulfamates) as well as the thiocyanates, tightly bind to the Zn(II) ion within the enzymatic active site, thus interrupting the carbon dioxide hydration cycle [1,2,12]. Although the sulfonamides are in clinical use as CAIs for almost 70 years they usually are associated to various side effects, which among others include hypersensitivity [1,12,13]. In the search for new and more effective drug candidates, natural products are indeed of particular interest. Up to now coumarins [18], polyamines, [19] and phenols [20,21] were revealed as interesting alternatives. The coumarins acts as prodrugs, [18] the simple phenols and polyamines anchor to the Zn(II)-bound water or hydroxide ion [19,20,21]. These different modes of action remarked many structural differences occurring between the CA isoforms and never explored before, thus paving the way for the design of new CAIs with high isoform selectivity and of particular interest for the future development as drug leads.

All the above considerations are of particular interest, as selective CA targeting is the crucial aspect for the successful pharmacological treatment of diseases in which such enzymes are involved. Besides the use of CAI-based drugs for the treatment of glaucoma, oedema and altitude sickness, CAs are established targets for the prognosis and treatment and of hypoxic tumors [22]. 

To date the hCA IX monoclonal chimeric antibody girentuximab (Rencarex^®^), and its iodo radiolabeled derivative (Redectane^®^) are marketed for both the treatment of renal cell carcinoma (RCC) and for imaging purposes respectively. Recent advances account for the introduction in phase I clinical trials of the primary sulfonamide small molecule **SLC-0111** for the treatment of metastatic solid tumors in patients showing positive response to hCA IX and XII markers [23]. hCAs are also valid therapeutic targets for the treatment of osteoporosis [24], central-nervous-system (CNS) affecting diseases such as Alzheimer or epilepsy [17,25,26,27], as well as for the treatment of obesity [28,29]. A research stream of particular interest is also represented by development of new antiinfectives by means of the selective targeting of the CAs expressed in bacteria, fungi and protozoa which represent the etiologic agents of various infectious diseases [30,31]. The development of new and more efficient antiinfectives is a field of prior interest in medicinal chemistry since many resistant bacterial strains are seriously jeopardizing the currently used pharmacological drugs. In this context natural products constitute a remarkable source of highly promising lead compounds for future development as next generation drugs.

### 1.2. Plant Phenolics 

Phenols and polyphenols constitute one of the largest and most ubiquitous classes of secondary metabolites in the plant kingdom. They are widely distributed in the Pteridophyta and Spermatophyta taxa (Gymnospermae and Angiospermae), whereas they are less common in bacteria, fungi and algae [32].

**Figure 1:**
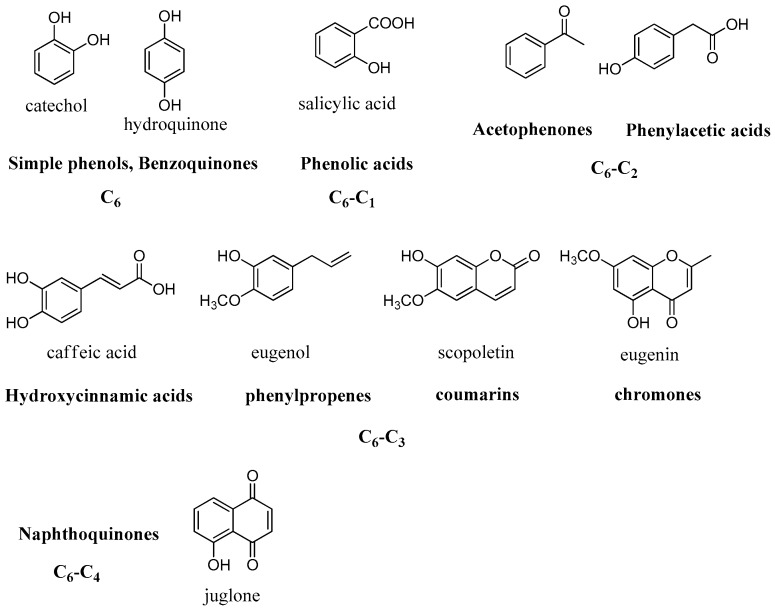
Main examples of phenols according to the classification of Harborne [32].

Simple phenols comprise structures with one aromatic ring, substituted with one or more hydroxyl moieties (Figure 1). Most phenols of natural origin have at least two hydroxyls in their structure and are generated biosynthetically from the shikimate (phenylpropanoid) pathway (C_6_-C_3_) through a series of reactions including decarboxylation, dehydration, and oxidative cleavage [33,34,35].

**Figure 2:**
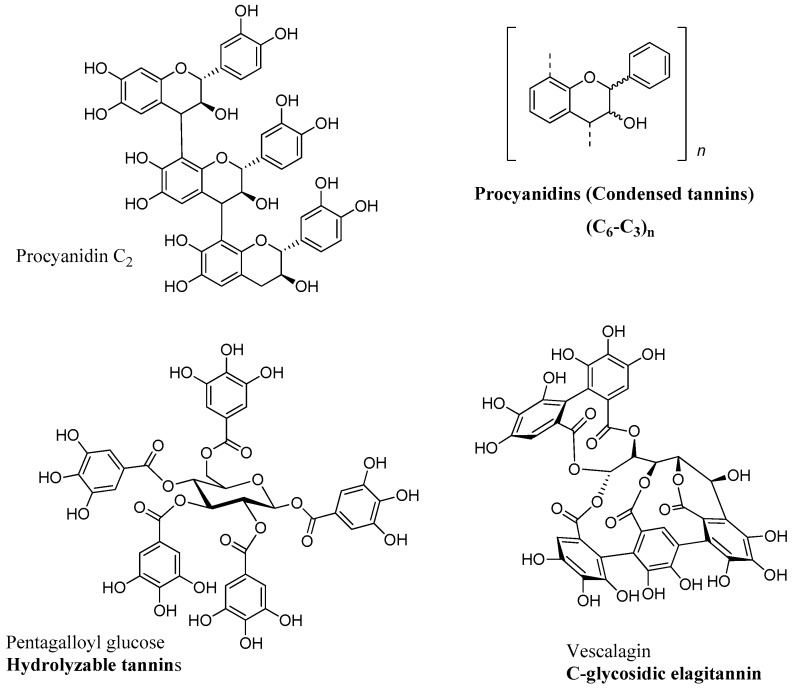
Examples of condensed (procyanidins), hydrolysable tannins and elagitannins.

**Figure 3:**
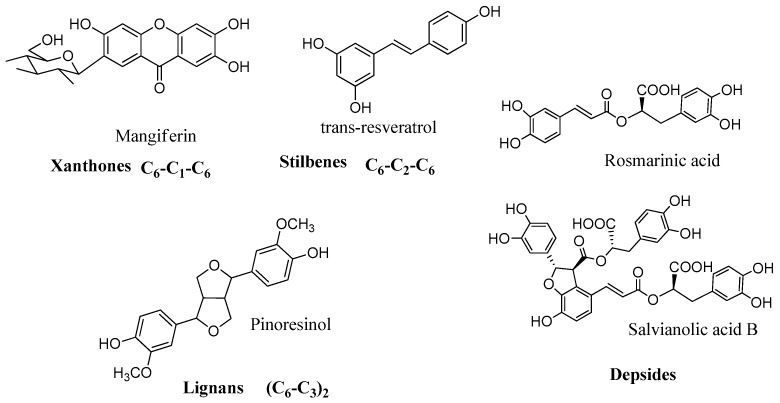
Some examples of polyphenolic classes.

Polyphenols have at least two phenolic rings in their structure and are characterized by a wide structural diversity (Figure 2 and Figure 3). They derive from the shikimate (phenylpropanoid) pathway, the polyketide (acetate) pathway or from a combination of both biosynthetic routes. Historically the term polyphenol referred to tannins (condensed, hydrolysable and elagitannins, Figure 2), which are complex polyphenolic compounds of polymeric nature with molecular masses ranging from 500 to 4000 Da, capable of interacting and binding with proteins. This property of tannins to act as protein precipitants has been exploited since antiquity in the processing of animal skin to leather [33,36,37].

**Figure 4:**
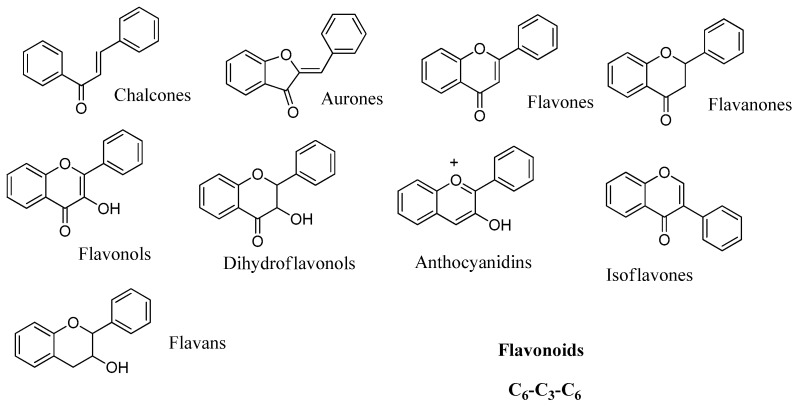
Basic flavonoid structures.

With the advent of chromatographic techniques and the development of phytochemistry, the term polyphenol was expanded to include a wider range of molecules with at least two phenolic rings in their skeleton: xanthones, stilbenes and stilbenoids, depsides, phenylpropanoid derivatives, lignans and lignin (Figure 3) and, most importantly, flavonoids (Figure 4), which are by far the biggest group [32] of polyphenolic compounds.

More than 8000 flavonoids and derivatives thereof have been reported to date [38]. The term flavonoid refers to yellow (*flavus* = yellow) pigments which in their classical form incorporate a benzo[γ]pyrone system. However, under the same group several biogenetically relative secondary metabolites are comprised including the yellow pigments aurones and chalcones, the colorless flavanones and the red/blue anthocyanidins (Figure 4).

In plants phenolics play a key role in many vital physiological processes, such as lignification, pigmentation of flowers and fruits, pollination, as growth factors, and they also govern plant responses towards environmental stress, such as protection from excess UV radiation, nutrient deficiency, drought stress, and most importantly they act as chemical defense against herbivores, insects and microbial pathogens [36,37]. For humans, plant phenolics are important constituents of many edible and medicinal plants and widely used in the food industry as flavorings, antioxidants and antibacterial agents [39,40]. Phenols and polyphenols have attracted great interest due to their wide presence in our daily diet and most importantly due to their antioxidant properties. Many efforts have been done to estimate the daily intake in plant phenolics. Humans seem to have adapted their enzymatic systems and only a small portion of the phenolic content in foods reaches the tissues unaltered [41]. Although the relationships between food phenolics and health are not fully understood, epidemiological studies in humans demonstrate that consumption of food rich in polyphenolic constituents might be beneficial especially for some age-related disorders, such as cardiovascular and neurodegenerative diseases, type II diabetes and cancers [41,42,43].

The ability of plant phenolics to interact with a plethora of targets and cause such a broad range of activities should be attributed to the physicochemical properties of the main structural unit, the phenolic ring [41]. The phenolic moiety has an amphiphilic character. The presence of the hydrophobic planar aromatic ring is responsible for hydrophobic interactions (π-stacking), whereas at the same time the polar hydroxyl groups can participate in hydrogen bonding. This dual behavior allows these molecules to bind to the amino acid residues of several proteins, enzymes or receptors. Furthermore the occurrence of ortho-hydroxyl groups renders them excellent antioxidant agents and chelating factors. These properties render plant phenolics as excellent candidate molecules to study the inhibition of metalloenzymes. 

One such an enzyme is the family of the carbonic anhydrases (CAs, EC 4.2.1.1) which is the subject of the present review. Simple phenols and polyphenols of natural origin are discussed, along with their synthetic derivatives. The inhibition mechanisms and possible structure activity relationships are also presented. Since coumarins are discussed in another article of this special issue they are not included in the present one. Only reports on pure compounds were taken into consideration. This is an update of previous reviews [44,45].

## 2. Phenols and Polyphenols as Effective Carbonic Anhydrase Inhibitors

### 2.1. Inhibition Mechanism of Phenol

**Figure 5:**
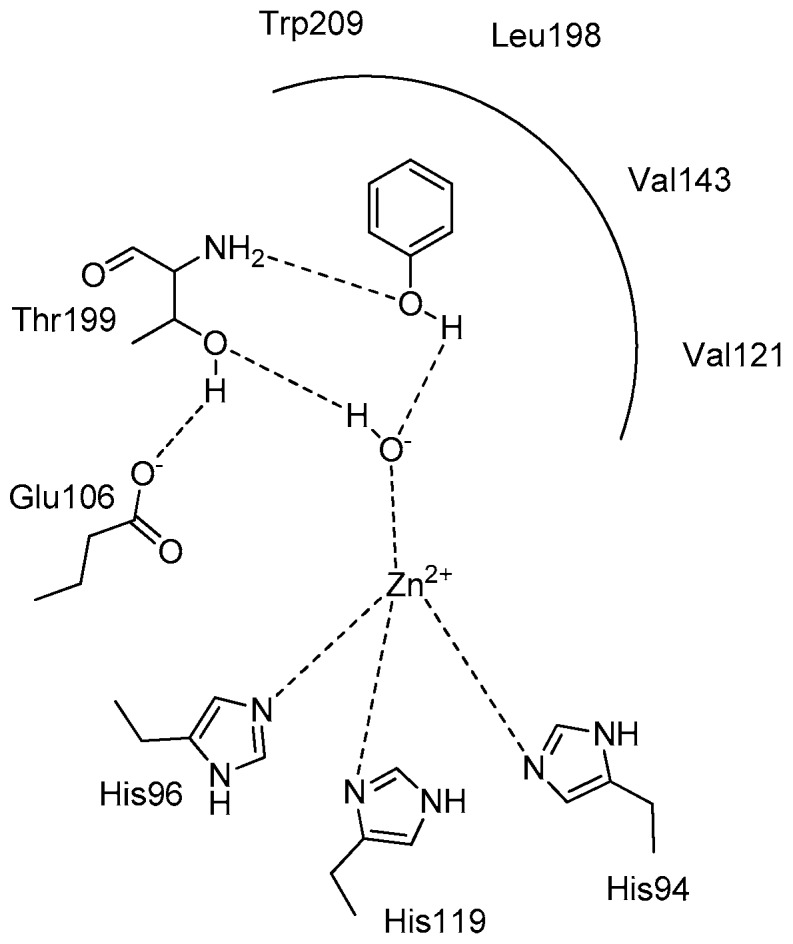
Schematic representation of the binding mode of phenol within the hCA II active site [48].

Phenol, the simplest member of this family of compounds was reported to be a competitive inhibitor of human (h) Carbonic Anhydrase II (hCA II) [46,47]. The X-ray crystal structure of its adduct with hCA II was studied and revealed the binding of two phenol molecules to the enzyme. The first one was located in a hydrophobic patch about 15 Å away from the catalytic zinc ion thus it is likely that this binding does not interfere with the enzymatic catalytic activity. However, the second phenol molecule was found within the active site and showed a new binding mode. Indeed, this molecule did not coordinate to the zinc ion, but was anchored to the active site through two hydrogen bonds of the OH moiety with the zinc-bound water/hydroxide ion and the NH amide of Thr 199. Furthermore, the aromatic ring was found to lie in a hydrophobic pocket of the active site, delimited by residues Val 121, Val 143, Leu 198, and 197 Trp 209, thus contributing to the complex stabilization [48] (Figure 5).

### 2.2. Simple Phenols 

**Figure 6:**
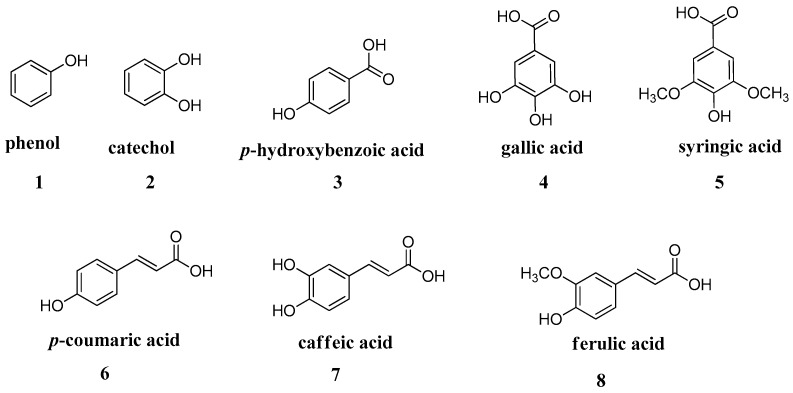
Some phenolic compounds as CAIs [45].

These findings opened the way for the exploration of other phenolic and polyphenolic natural products. In vitro kinetic studies of a series of phenols widely available on the market such as salicylic acid, resorcinol, *p*-coumaric, caffeic, ferulic, gallic acids, etc., showed that these are affective CA inhibitors in the low micromolar range (Figure 6) [45,46]. 

**Figure 7:**
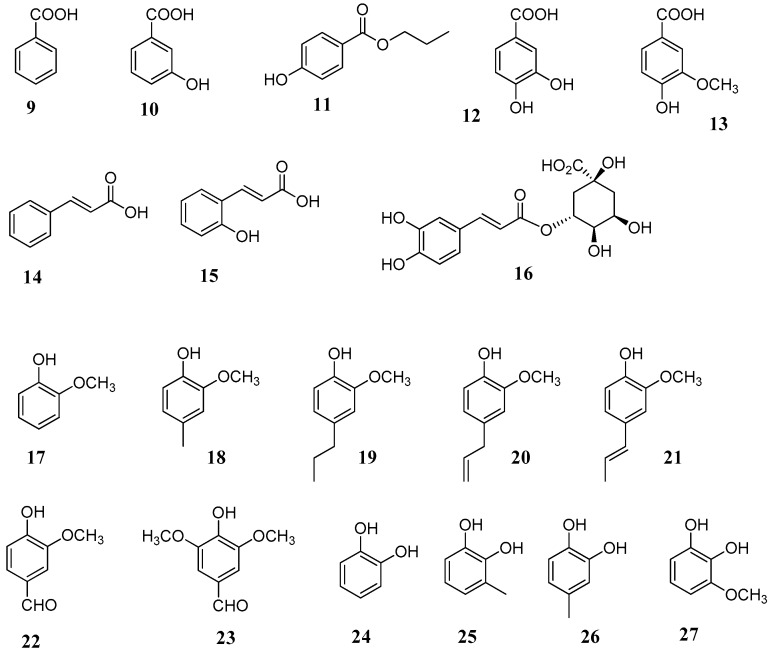
Structures of tested phenols: benzoic acid (**9**), *m*-benzoic acid (**10**), propylparaben (**11**), protocatechuic acid (**12**), vanillic acid (**13**), cinnamic acid (**14**), *o*-coumaric acid (**15**), chlorogenic acid (**16**), guaiacol (**17**), 4-methylguaiacol (**18**), 4-propylguaiacol (**19**), eugenol (**20**), isoeugenol (**21**), vanillin (**22**), syringaldehyde (**23**), catechol (**24**), 3-methylcatechol (**25**), 4-methylcatechol (**26**), 3-methoxycatechol (**27**) [45].

The presence of free hydroxyl groups at position 4’ in combination with the double bond in the phenylpropanoid skeleton, as in *p*-coumaric acid, clearly enhanced the inhibitory activity when compared to the simple phenol moiety. Similar results were obtained in a further study with a small group of monophenolic constituents, where *trans*-cinnamic (**14**), *o*-coumaric (**15**) and chlorogenic (**16**) acids were more effective than simple phenolic constituents such as benzoic acid (**9**) [45] (Figure 7 and Table 1).

In another study several derivatives of guaiacol and catechol (**17**–**27**) were considered for their CA inhibitory activity against human CA I, II, IX and XII isoforms [49]. Among the compounds tested in vitro were the volatile constituents eugenol (**20**), isoeugenol (**21**) and vanillin (**22**), which are major components of many aromatic herbs and spices such as clove, basil, anise seed, *Vanilla* sp., etc. All compounds showed micromolar inhibition potencies spanning between 2.2–10.92 μM with the exception of the catechol (Table 2). Among the tested phenolic derivatives, compounds 4-methyl-catechol (**26**) and 3-methoxycatechol (**27**) showed potent activity as inhibitors of the tumour-associated transmembrane isoforms hCA IX and XII with K_I_ values in the submicromolar range.

Combination of the phenolic moiety with other functional groups is a common strategy to obtain products with enhanced activity. For example, in the so-called “sugar approach” the incorporation of a hydrophilic moiety of a sugar permits to the molecule to anchor in a different manner to the enzyme cavity. Furthermore, it increases its selectivity towards the membrane bound CA isoforms over the cytosolic ones [50,51]. This is desirable since selectivity against specific hCA isoforms might reduce side effects.

**Figure 8:**
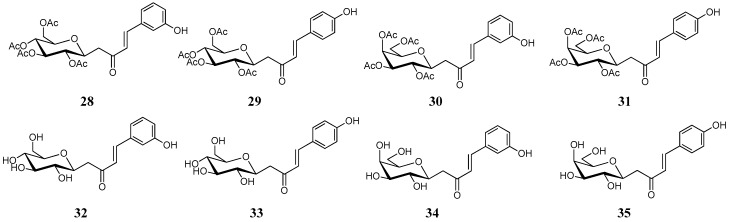
Structures of the cinnamoyl glycosides **28**–**35** [52].

In this concept, a series of synthetic *C*-cinnamoyl glycosides were synthetized and considered for the inhibition on twelve mammalian isoforms of carbonic anhydrase (Figure 8) [52]. These compounds combined two functional groups with known activity against the hCAs: the cinnamoyl and the glucose group. In order to avoid undesirable degradation, the *O*-glycosidic bond was replaced by the more stable *C*-glycosidic one. The *C*-cinnamoyl glycosides (**28**–**35**) were generally effective CAs, with inhibition constants in the low micromolar range against CA I, II, IV, VA, VB, VI, VII, IX, XII, XIII, XIV and ineffective inhibitors of CA III (Table 3).

**Figure 9:**
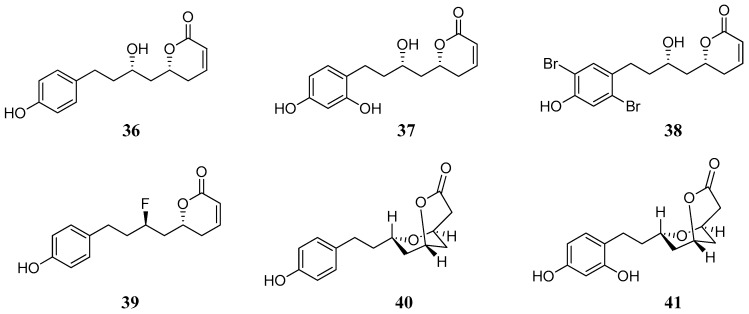
Structures and hCA inhibition data of dodoneine (**36**) and its analogues **37**–**41** by a stopped flow CO_2_ hydrase assay [53].

Another hybrid molecule recently investigated against several hCA isozymes [53] is Dodoneine **36**, one of the active principles of the medicinal plant *Agelanthus dodoneifolius* (DC) Polhilland Wiens. The plant is used in traditional medicine for the treatment of hypertension. Structurally **36** incorporates both the phenol and lactone (α-pyrone) moieties. Dodoneine showed to be selective in inhibiting the hCA isoforms I, III, IV, XIII and XIV with K_I_s in the range of 5.5–10.4 µM. CA I, III and XIII are cytosolic, CA IV is localized in plasma membrane and hCA XIV is a membrane bounded isozyme. Further in vitro and in vivo studies in rat aorta and vascular smooth muscle cells showed that hCA II, III, XIII and XIV were expressed in rat aorta while only the hCA isoforms III, XIII and XIV were expressed in smooth muscle cells. Thus it is reasonable to assume that dodoneine induced vasorelaxation by a dual mode of action including both block of the L-type calcium channels and inhibition of the hCA isoforms therein expressed [54]. A series of synthetic analogues of dodoneine (**37**–**41**), reported in Figure 9, were prepared and tested for their inhibition properties against the hCAs. Among them the bicyclic compound **40** which is normally found in the plant in small amounts and its hydroxylated derivative **41** and the fluorinated analogue **39** (Table 4). Compound **39** inhibited all hCA isoforms, while **41** inhibited selectively hCA III (K_I_ = 5.1 nM) and mCA XIII (K_I_ = 340 nM).

### 2.3. Polyphenols

**Figure 10:**
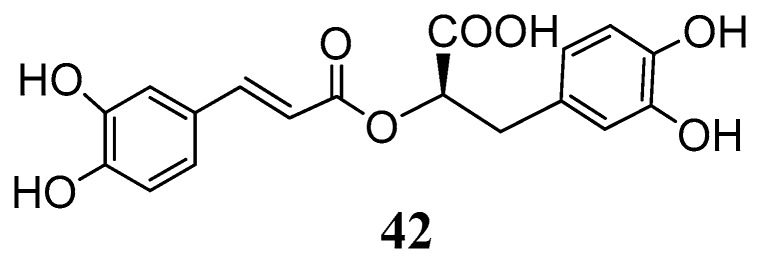
Structure of rosmarinic acid (**42**).

Recently rosmarinic acid (**42**) was reported to be a potent inhibitor of the hCA I and II isoenzymes at low micromolar range with K_I_ values of 86.0 µM and 57.0 µM, respectively (Figure 10) [55]. Rosmarinic acid is a powerful antioxidant found in many aromatic, medicinal and culinary herbs of the Lamiaceae family, such as rosemary, sage, oregano and thyme [56]. Chemically it belongs to the class of depsides, or the so-called labiataetannins [41], which are obtained by condensation of caffeic acid units or by combination of caffeic acid with 3,4-dihydroxyphenyl lactic acid, to form dimers, trimers and tetramers. 

**Figure 11:**
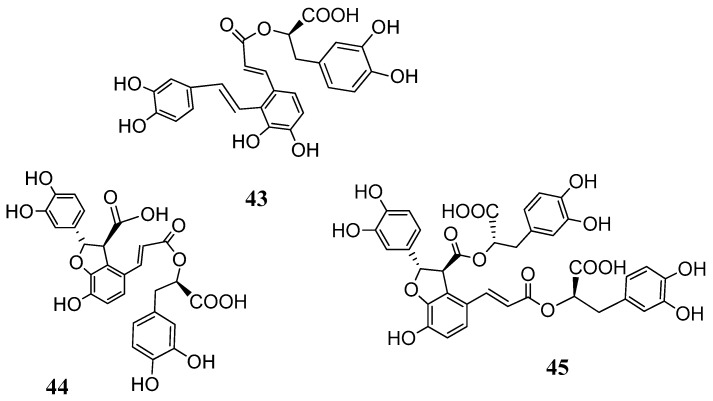
Structures of salvianolic acids **43**–**45** from *Salvia miltiorrhiza*.

Based on these results further investigations were carried out with the salvianolic acids **43**–**45** (Figure 11) isolated from *Salvia miltiorrhiza* (Lamiaceae) [57,58]. *Salvia miltiorrhiza* or Danshen is one of the most important herbal medicinal drugs in the Chinese Traditional Medicines (TCM) to treat bleeding disorders (e.g., menstrual bleeding) and blood stasis [59]. Salvianolic acids are together with tanshinones (lipophilic, diterpenic quinones) the main active principles of the herbal drug. These hydrophilic derivatives, abundant in the roots of the plant, incorporate three to four units of caffeic/3,4-dihydroxyphenyl lactic acids. They have attracted interest due to their protective roles in CNS neuronal injuries and degeneration. The main mechanism involved in such processes is the decrease of reactive oxygen species (ROS) levels. Furthermore, there is evidence of the protective role of salvianolic acids in several cardiovascular diseases, osteoporosis, liver fibrosis and hepatic failure [59,60,61].

The CAs inhibition studies reported in Table 5 clearly showed that depsides **43**–**45** were ineffective hCA I and weak hCA II inhibitors. On the other hand, they were inhibitors of the hCA IV with K_I_ values in the range of 62.2–102.1 nM and comparable to the standard drug acetazolamide **AAZ** (74.0 nM). Even if a proper structure–activity-relationship (SAR) consideration is difficult to be drawn it is noteworthy that the flexible and more containing catechol moieties salvianolic acids A and B (**43** and **45**) were better inhibitors when compared to the lithospermic acid **44**. Furthermore, salvianolic acids A and B were medium and high nanomolar inhibitors (K_I_ 39.8 and 453.6 nM of the tumor associated CA XII. By contrast, lithospermic acid (**44**) showed to be a very potent hCA XII inhibitor with a K_I_ value of 4.8 nM. The presence of ortho-hydroxyl moieties in these molecules, the possibility to release in situ caffeic acid or even the presence of free carboxyl groups are some of the structural characteristics that play an important role in the CA inhibitory activity. In any case such results put in evidence a new class of natural products which deserves further investigation in order to decipher a proper SAR correlation.

**Scheme 1:**
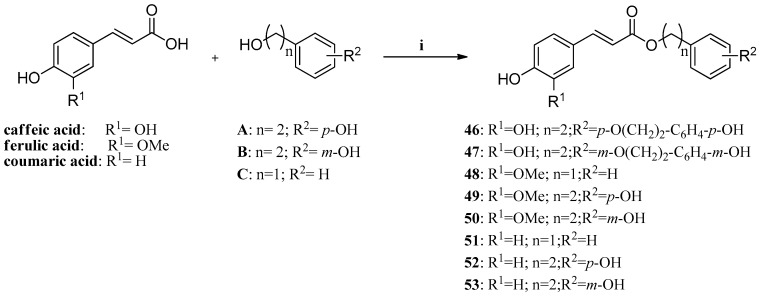
Synthesis of phenolic derivatives **46**–**53** *Reagents and conditions*: (i) THF, Ph3P, DIAD, 0 °C [62].

Another recent example is constituted by a series of phenolic acid esters (Scheme 1) incorporating caffeic, ferulic, and *p*-coumaric acid, and benzyl, *m/p*-hydroxyphenethyl- as well as *p*-hydroxy-phenethoxy-phenethyl moieties, which were investigated for their inhibitory effects against the mammalian isozymes of human (h) or murine (m) origin, hCA I–hCA XII, mCA XIII and hCA XIV [62]. These enzymes were inhibited in the submicromolar range by many of these derivatives (with K_I_s of 0.31–1.03 μM against hCA VA, VB, VI, VII, IX and XIV). The off-target, highly abundant isoforms hCA I and II, as well as hCA III, IV and XII were poorly inhibited by many of these esters, although the original phenolic acids were micromolar inhibitors. These phenols, like others investigated earlier, possess a CA inhibition mechanism distinct of the sulfonamides/sulfamates, clinically used drugs for the treatment of a multitude of pathologies, but with severe side effects due to hCA I/II inhibition.

**Scheme 2:**
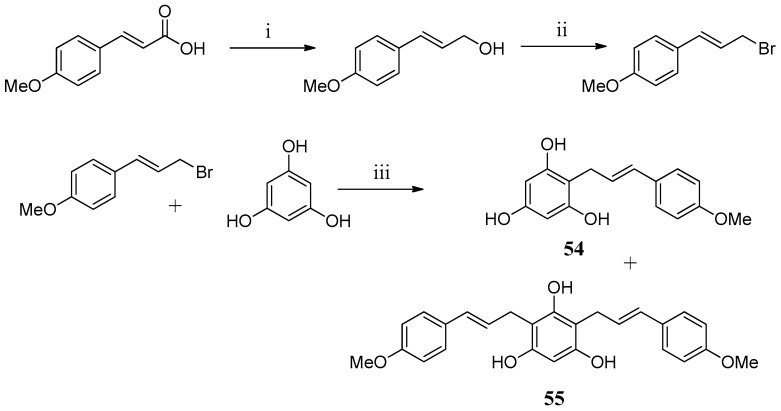
Synthesis of floroglucinol derivatives **54** and **55**. *Reagents and conditions*: NaBH_4_, EtOH, 0 °C, 30 min, 98%; (ii) PBr_3_, diethylether, 0 °C, 1 h, 85%; (iii) NaH, THF, r.t., 17 h, 26%.

In an attempt to find new chemotypes, two floroglucinol derivatives **54**, **55** were prepared and tested against hCA I and hCA II (Scheme 2). Unfortunately, none of the compounds proved to be active [63].

### 2.4. Flavonoids

**Figure 12:**
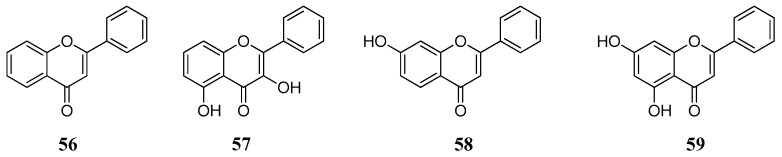
Structures of compounds **56**–**59**.

Data of the interaction of flavonoids with CAs indicate that these may be another category of CAIs of interest, which can be used as leads for generating more potent CAIs. Preliminary test of a small number of basic flavonoid structures **56**–**59** (Figure 12) against hCA I, II, IX and XII showed that these act as low micromolar inhibitors and their effect is independent of the incubation time [64]. This fact indicates that flavonoids inhibit CAs probably through a mechanism similar to the one of phenol rather than hydrolysis of the chromenone as in the case of coumarins.

**Figure 13:**
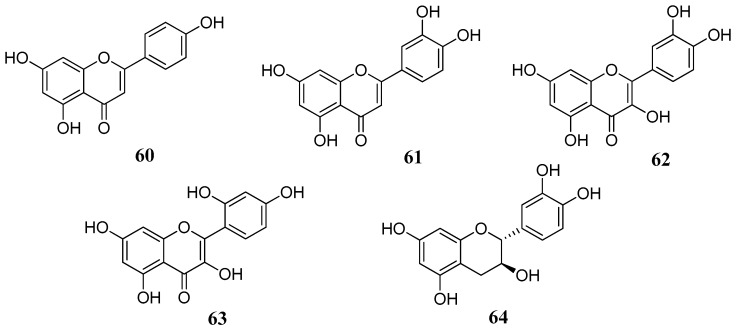
Structures of compounds **60**–**64**.

This hypothesis was further confirmed in several studies including flavonoids lacking the C-4 carbonyl group. A series of flavonoids **60**–**64** with different substitution patterns were investigated for their inhibitory effects against four α-CA isozymes from human and bovine (hCA I, hCA II, bCA III, hCA IV) tissues: the flavones apigenin (**60**) and luteolin (**61**), the flavonols quercetin (**62**) and morin (**63**) and the flavan-3-ol, catechin (**64**) (Figure 13) [65]. As it can be observed from Table 6, flavonoids **60**–**64** were quite effective, similar to the standards used. The four hCA isozymes showed quite diverse inhibition profiles with these compounds suggesting that the type of flavonoid and/or substitution pattern are important for the activity. As briefly outlined above, the C-4 carbonyl group is not so important for the inhibition, as shown by the example of catechin (**64**). This is another indication that the inhibition mechanism does not proceed via hydrolysis (CA esterase activity). Instead, the presence of one or two neighboring hydroxyl groups is essential, as shown by the example of morin **67** which is the less potent compound compared to quercetin **62** or luteolin **61**. The latter, **61** showed the most effective inhibitory activity with K_I_s in the low micromolar range. 

**Figure 14:**
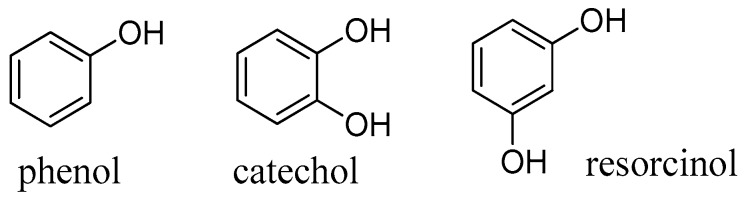
Structures of basic phenol rings: phenol, catechol and resorcinol.

Comparison with the relative monophenols, catechol, phenol and resorcinol (Figure 14), which were considerably less active, suggests that also the ring A of flavonoids may be involved in the inhibitory mechanism. Whether this is due to a different mechanism or simply stronger binding to the enzyme cavity (e.g. by further hydrogen bonds) is to be investigated in the future.

**Figure 15:**
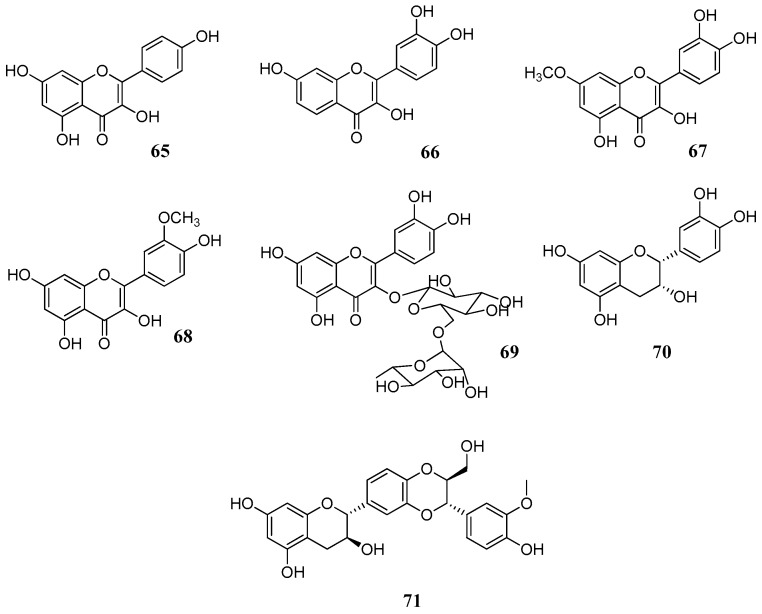
Structures of compounds **65**–**71** [46,67].

Two further studies [46,67] with several flavonoids **60**, **62**, **64**–**71** (Figure 15, Table 7 and Table 8) against human CA I, II, IV, VI and bovine CA III isoforms gave further evidence that the catechol moiety on C-2 enhances the activity, as demonstrated in the case of fisetin (**66**), rhamnetin (**67**) and rutin (**69**). Although a comparison between results of Table 7, Table 8 and Table 9 is not feasible due to the different enzymatic assays used and to different enzyme purities, it can be observed that catechin (**64**) and epicatechin (**70**) have similar activities despite different stereochemistry on C-3. Similarly, the hydroxyl group of C-3 seems to be of little importance for the activity. This is evident if we compare luteolin (**61**) with quercetin (**62**) (Table 6) and apigenin (**60**) with kaempferol (**65**) (Table 7). Therefore, the keto-enol system apparently does not take part in the inhibition mechanism. The better inhibitory activity of fisetin (**66**) and rhamnetin (**67**) should be attributed to structural differences in ring A. Interestingly, rutin (**69**) compared to flavonol aglycones such as quercetin (**62**) or rhamnetin (**67**) is more active suggesting that sugars may play an important role, as discussed previously. 

**Figure 16:**
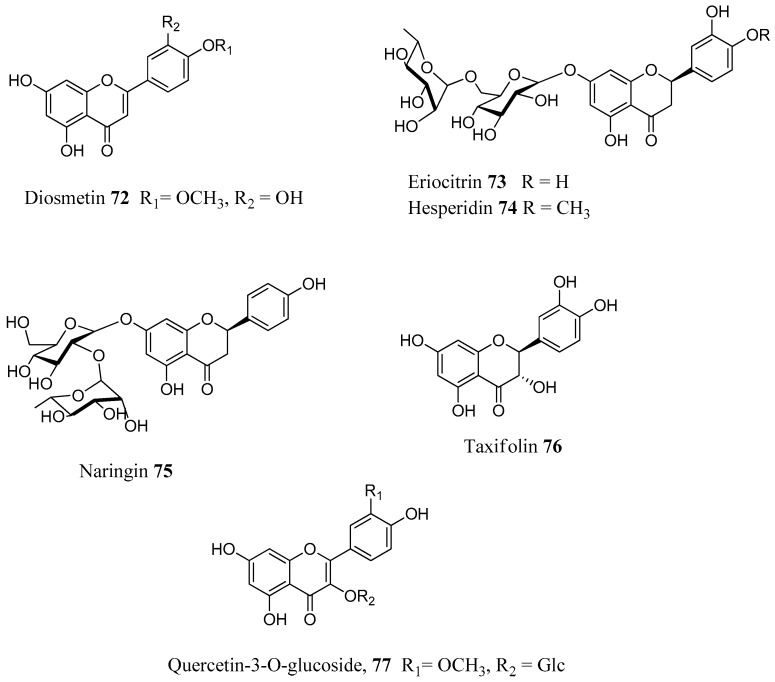
Structures of compounds **72**–**77** [69].

A possible explanation of the way that flavonoids interact with the CA active site was given in a very interesting docking study by Gidaro et al., [69]. In this work a structure-based virtual screening was performed against five carbonic anhydrase isoforms using, as a ligand library, natural components of *Citrus bergamia* (Bergamot) and *Allium cepa* var. *tropea* (red onion). Both plants are edible and part of the Calabrian (Italy) diet. By use of virtual screening, the flavone apigenin **60** and the flavanone glycoside eriocitrin (eriodictyol-7-*O*-rutinoside) **73** (Figure 16) were identified together with other eight flavonoids. In vitro tests against CA I, II, VA, IX, and XII isoforms showed that eriocitrin was the best hCA VA inhibitor with a K_I_ value of 0.15 μM. On the contrary, the computational prediction failed for apigenin. Docking studies revealed that eriocitrin probably binds by both the catechol and rutinoside portions. The catechol moiety is linked by additional H-bond interactions with the enzymatic residue Thr 199 located in the catalytic site, while the rutinose group connects by an extended H-bond network with several external mCA VA amino acid residues (Gln 67, Glu 69, Gln 92, Asp 171) at the entrance of the binding pocket. Apigenin instead, which was not as active as predicted, seems to interact only by its ring B which is involved in the zinc coordination into the mCA VA active site.

**Figure 17:**
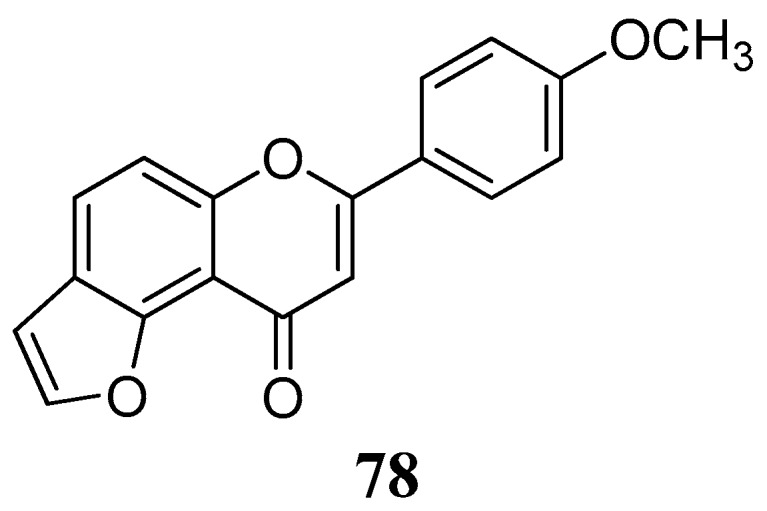
Structure of furanoflavonoid **78**.

The lipophilic furanoflavonoid **78** from the bark of *Millettia ovalifolia* (Fabaceae) was considered for inhibition of the cytosolic form of the bovine carbonic anhydrase-II (Figure 17) [70]. Despite the lack of free hydroxyl groups in its structure it displayed significant inhibitory activity with IC_50_ value of 17.86 ± 0.07 μM (vs. zonisamide, 1.86 ± 0.03). 

**Figure 18:**
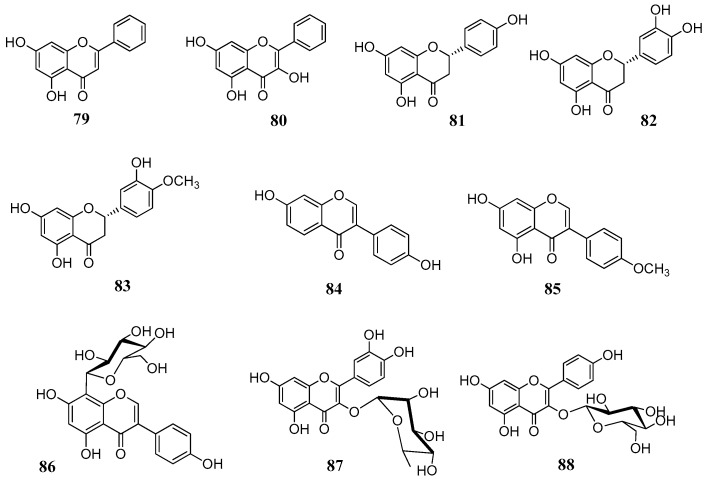
Structures of flavone **79**, flavonol **80**, flavanones **81**–**83**, isoflavones **84**, **85**, isoflavone glucoside **86** and flavonol glycosides **87**–**88**.

**Figure 19:**
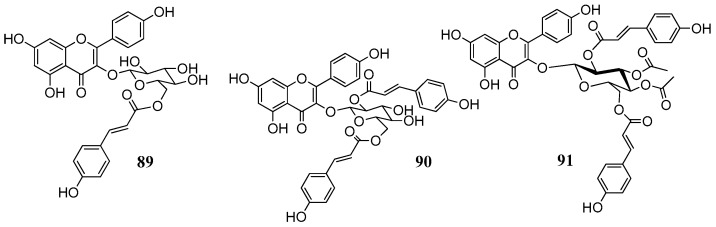
Structures of tested acylated flavonol glycosides **89**–**91** (isolated from *Quercus ilex*) [71].

The above reported results spurred us to investigate a larger number of flavonoids against a line of human CAs. In total, 18 flavonoids (Figure 18) were selected both from commercial sources and lab isolates [71]. The selection of the compounds was done on the basis of the flavonoid subgroup and the substitution pattern. Flavones, flavonols, flavanones, isoflavones and some glycosylated derivatives were considered for assays. Chrysin (**79**) is one of the main flavonoids of *Passiflora incarnata*, a medicinal plant used phytotherapy as a mild sedative and anxiolytic [72]. It is a flavone without any substitution of ring B. The flavone luteolin (**61**), the two flavonols kaempferol (**65**) and isorhamnetin (**68**), as well as the flavonol glycosides quercetin-3-*O*-glucoside (**77**), quercetin-3-*O*-rhamnoside (**87**) and kaempferol-3-*O*-glucoside (**88**) are ubipresent flavonoids in most vegetables and fruits daily consumed, while galangin (**80**) is present in honey and propolis [73,74]. Among the flavonoids considered were the flavanones naringenin (**81**), eriodictyol (**82**) and hesperitin (**83**), which are abundant in edible *Citrus* spp. (e.g. orange, mandarin) and possess antioxidant, anti-inflammatory and antitumoral properties [75]. The isoflavones **84**–**85** are abundant in legumes, soy and show potent estrogenic activity by interacting with the estrogenic receptors [76,77]. Puerarin (**86**) is the main active principle of *Pueraria lobata* root (Fabaceae) and it used in clinical practice in China to treat cardiovascular diseases, cerebrovascular disorders, Parkinson’s disease, Alzheimer’s disease, diabetes and diabetic complications [78]. The flavonols **89**–**91** were isolated from leaves of *Quercus ilex* (Fagaceae) and belong to the group of acylated flavonoids (Figure 19). Since they combine the flavonol nucleus and the coumaric acid moieties, they were selected with the aim to see whether we would have an additional inhibitory effect on the CAs. For the in vitro inhibitory activities five physiological relevant hCA isoforms were considered: the cytosolic I, II, VII and the membrane bound CA IV and XII. Results are displayed in Table 10.

Although a SAR is difficult, some general remarks can be made. As can be observed from Table 10, none of the compounds considered here show a significant inhibitory activity against the cytosolic hCA I, but rather showed selectivity against isoforms hCA IV and hCAV II. Concerning the inhibition of hCA II, the presence of ortho-hydroxyl groups on ring B seems to be important for the activity as long as the double bond in ring C remains intact. This is shown comparing luteolin with eriodictyol and taxifolin. Instead, glycosylation of the C-3 hydroxyl enhances the activity, as demonstrated by Kaempferol**-**3-*O*-glucoside which was the most active of all tested compounds with a K_I_ of 168 nM.

Results were quite surprising regarding the inhibition hCA IV. The flavanones naringenin, eriodictyol and hesperitin, lacking the C_2-3_ double bond as well as the glycosides **77**, **87**, **91** were quite effective against the with K_I_ values close to the standard CAI acetazolamide (74.0 nM). Especially comparing the activity of compounds **65**, **89**–**91** it could be suggested that the optimum activity of compound **91** is not due to the flavonoid scaffold but to the acetyl/coumaric functionalities which may hydrolyze and released in situ. This hypothesis requires further investigation. 

The kinetic data on the cytosolic hCA VII showed that several of the investigated compounds were low nanomolar inhibitors of the hCA VII with K_I_ very close to the standard acetazolamide. Among the investigated constituents are the flavone luteolin (with *ortho*-hydroxyl groups) the flavonol isorhamnetin, the flavanones naringenin, eriodictyol and hesperitin, the isoflavone daidzein, the quercetin flavonols **77** and **87** and the acylated glycosides **89** and **90**. With the exception of catechin, this is the first time that flavonoids are tested against this particular hCA isoform [68]. The heterogeneity of constituents does not permit any SAR conclusion or it is possible that some other common structural feature may be responsible for the inhibition, such as ring A or combination of rings A and B. Similarly, the inhibition data on the tumor associated hCA XII isoform (Table 10) showed a complex and different SAR. Generally most of the substances were high nanomolar inhibitors with K_I_ values spanning between 515.0 and 134.3 nM. The best inhibitor evidenced was Kaempferol-3-*O*-glucoside with a K_I_ value of 4.9 nM. The results in both hCAV II and XII show that flavonoids merit further attention and warrant future investigation to comprehend the mechanism of action in order to design new, selective inhibitors.

**Figure 20:**
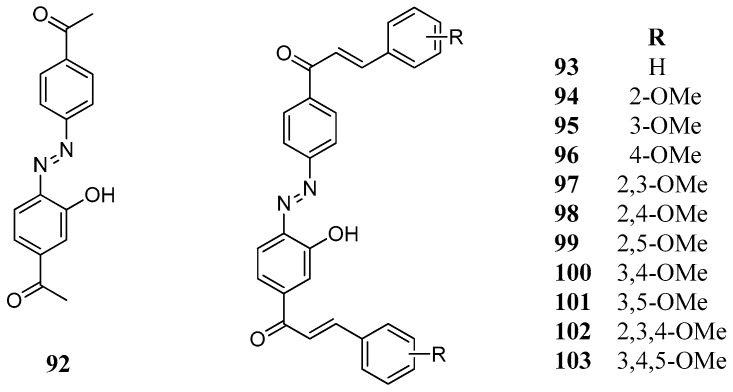
Structures of compounds **92**–**103**.

A series of synthetic bischalcones **92**–**103** bearing a C–N=N–C linkage has been prepared and tested against human hCA I and II (Figure 20) [79]. Most of the compounds had one to three methoxy groups as substituents on their phenolic rings. Compounds **93**, **97**, **101**, and **103** were among the best inhibitors (Table 11). Furthermore, similarly to AZA, some of the investigated bischalcone compounds act as competitive inhibitors with 4-NPA as substrate, that is, they bind to the same regions of the active site cavity as the substrate. However, the binding site of 4-NPA itself is unknown, but it is presumed to be in the same region as that of CO_2_, the physiological substrate of this enzyme.

### 2.5. Phenols and Polyphenols as Effective Mycobacterial CA Inhibitors

The discovery of the α-, β-, and η-families of carbonic anhydrase in parasitic bacteria, fungi protozoa and helminths with different structure than human CAs has created new opportunities in the search for new drugs against pathogens [29]. Since many pathogen carbonic anhydrases belong to different enzyme families, which are either absent in humans (like β- and η-CAs) or have marked structural differences with the human isoforms they consist attractive targets for the development of new anti-infective agents in terms of selectivity and efficacy. Given the continuous development of resistance of several pathogens to the existing therapeutic schemes, CA inhibition represents a new, complementary mechanism of action worth of investigation. Among the most investigated CAs, are the β-CAs from *Helicobacter pylori*, *Brucella suis* and *Mycobacterium tuberculosis,* which have been cloned and characterized [80,81]. Other β-CAs which have been intensely studied are the CAs from the fungal strains *Candida albicans*, *Candida glabrata* and *Cryptococcus neoformans*: CaNce103 and CgNce103 (from *C. albida* and *C. glabrata*, respectively) and Can1, Can2 from *C. neoformans*. In these pathogens CAs regulate the physiological concentrations of CO_2_/HCO_3_. Both fungal strains are adapted to extremely low concentrations of CO_2_. However, once the infection occurs, elevated concentrations of CO_2_ promote distinct structural and developmental procedures which influence the survival, virulence and pathogenesis of these fungi [82]. 

**Figure 21:**
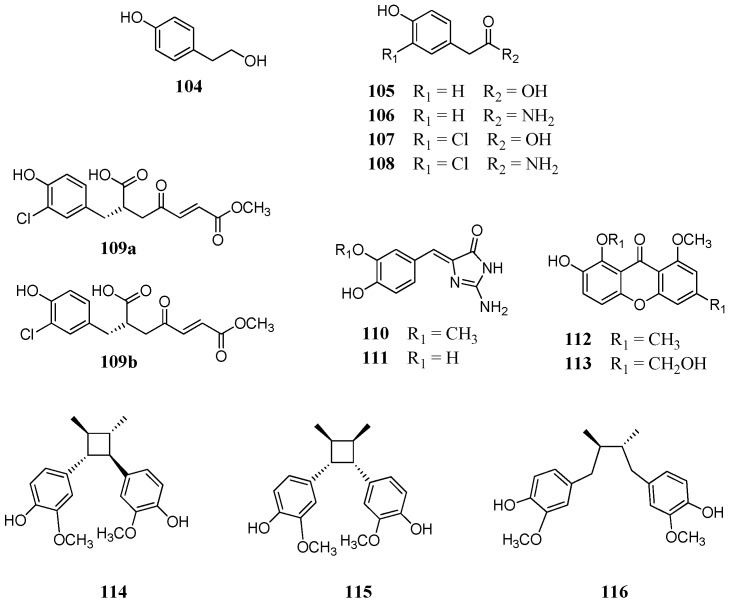
Structures of compounds **104**–**116**.

**Figure 22:**
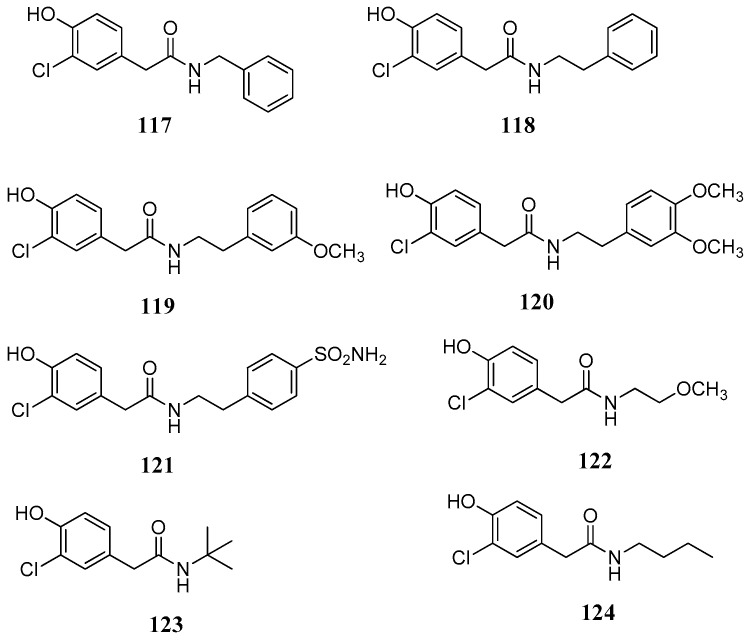
Structures of synthetic derivatives **117**–**124**.

The first evidence that phenols can inhibit pathogenic CAs came from the screening of a natural product library [83] against β-CAs from *M. tuberculosis*, *C. albicans*, and *C. neoformans*. Screening of libraries of plant extracts and isolated constituents, although time consuming, is a valuable tool in the exploration of new scaffolds in order to discover new chemotypes to combat diseases. The Queensland Compound Library reported by Davis et al., [83] comprised a wide range of natural and synthetic products incorporating the phenol group. Overall 21 constituents **104**–**124** were selected and tested (Figure 21 and Figure 22) including eight fungal (compounds **104**–**109a**, **112**, **113**), two ascidian (compounds **107**, **108**), and three plant (compounds **111**–**113**) secondary metabolites. The tested structures were diverse comprising a series of simple mono- or disubstituted phenols **104**–**108**, (−)-xylariamide A (**109a**) and its synthetic enantiomer (+)-xylariamide A (**109b**), polyandrocarpamine A (**110**), polyandrocarpamine B (**111**), xanthones **112** and **113**, endiandrin A (**114**), endiandrin B (**115**) and (−)-dihydroguaiaretic acid **116**. The synthetic phenolic compounds **117**–**124** (Figure 22) were eight secondary amides inspired by the fungal metabolite **108**. 

Inhibition studies over human CAs were carried out in parallel in order to assess the selectivity of the tested compounds. As it can be observed from Table 12, several phenolic natural products were selective inhibitors of mycobacterial and fungal β-CAs. 

**Figure 23:**
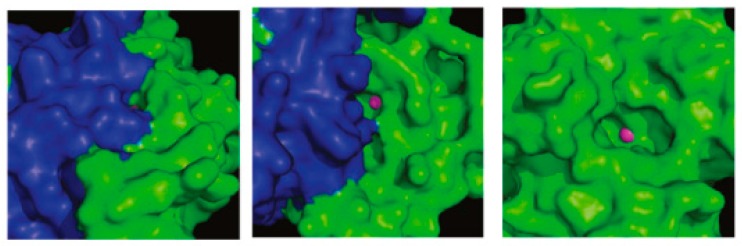
Comparison of accessible surface volumes of the active sites of *M. tuberculosis* Rv1284 (left, PDB accession code 1ylk), *C. albicans* Nce103 (middle, PDB accession code 3eyx), and human CA II (right, PDB accession code 3NB5). The individual monomers of the dimeric β-CAs are colored in green and blue, respectively. The catalytic zinc ion is shown in magenta. The figure was prepared with PyMol [84].

Among the natural products tested the best inhibitors was (−)-dihydroguaiaretic acid (**116**) which inhibited β-CA in the submicromolar range with up to 495-fold selectivity over hCA I and 371-fold selectivity over hCA II. From the library of synthetic derivatives, compound **108** was a low micromolar inhibitor of the fungal CAs and displayed 130- to 280-fold selectivity over the two human CAs. These compounds were the first non-sulfonamide inhibitors that displayed β- over α-CA enzyme selectivity. This selectivity could be explained if the different structure of α- and β-isozymes is taken into consideration. Although both enzymes contain a zinc ion in the active site, they have several differences. Unlike α-CAs, the β-class of CAs exist as dimers (Figure 23). Whilst the catalytically important residues in the active site of β-CAs are provided exclusively from one monomer, access to the catalytic center may be constituted by residues from both monomers leaving a cleft of limited space for the inhibitor to enter. Therefore, it seems unlikely that the phenolic hydroxyl groups of the tested constituents directly interact with the active site zinc of the pathogen CAs, especially for the spacious one **116**. It was suggested that the compounds might cause either monomerization or considerable conformational changes in the access area to the active site. To prove this concept crystallization of the adduct, and X-ray analysis is necessary. Unfortunately none of the tested compounds gave crystals with Can2, Nce103, Rv3273, and Rv1284 enzymes. 

**Figure 24:**
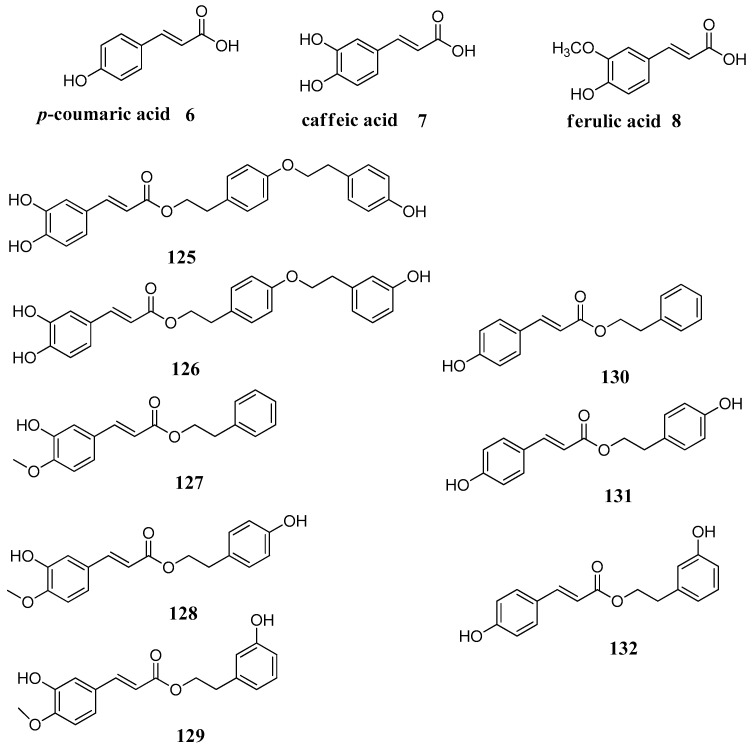
Structures of compounds **6**–**8** and derivatives **125**–**132**.

A series of phenolic acids **6**–**8** and some of their esters **125**–**132**, derivatives of *p*-coumaric (**6**), caffeic (**7**) and ferulic acid (**8**), was investigated for the inhibition of three β-carbonic anhydrases (CAs, EC 4.2.1.1) from the pathogenic bacterium *Mycobacterium tuberculosis*, Rv1248, Rv3588 and Rv3273 β-CAs (Figure 24) [85]. Some of these compounds were low micromolar inhibitors of the pathogenic enzymes and they did not show inhibitory activity against the human widespread cytosolic isoforms CA I and II. As it can be observed from Table 13, simple phenolic acids **6**–**8** are active against Mtb β-CAs but suffer of low selectivity. The presence of additional phenolic rings in the structure makes the compounds more selective as it increases the ratio of β- over α-CA inhibitory activity. This is possibly due to additional interactions with the dimeric structure of the β-CA. 

**Figure 25:**
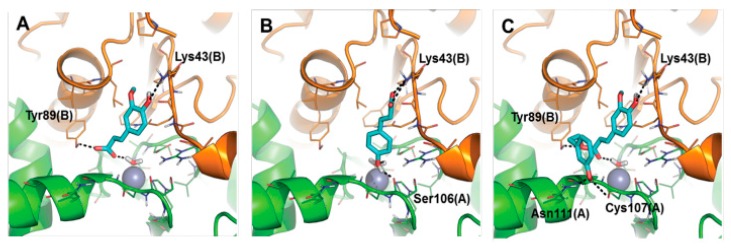
Predicted binding mode of 2 (panel **A**), 3 (panel **B**) and 8 (panel **C**) towards the active site of Rv3588 β-CA from Mtb. Monomer A is colored green, monomer B is colored orange. Small molecules are shown as cyan sticks. Residues within 5 Å from the Zn(II) ion and those in contact with the inhibitors are shown as lines. H-bond interactions are highlighted by black dashed lines. Residues involved in H-bonds are labeled. The catalytic Zn(II) ion is shown as a grey sphere [85].

**Figure 26:**
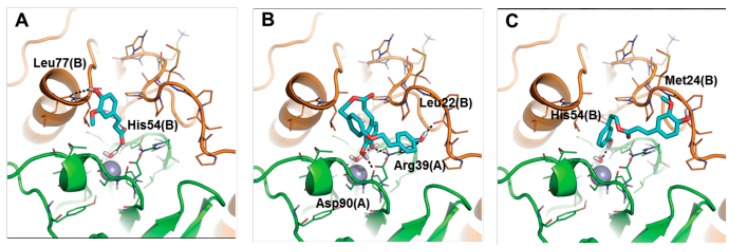
Predicted binding mode of 2 (panel **A**), 4 (panel **B**) and 8 (panel **C**) towards the active site of Rv1248 β-CA from Mtb. Monomer A is colored green, monomer B is colored orange. Small molecules are shown as cyan sticks. Residues within 5 Å from the Zn(II) ion and those in contact with the inhibitors are shown as lines. H-bond interactions are highlighted by black dashed lines. Residues involved in H-bonds are labeled. The catalytic Zn(II) ion is shown as a grey sphere [85].

Unfortunately it was not possible to obtain good quality crystals which would permit the study of the adduct by X-ray crystallography. To overcome this problem, the binding mode of these inhibitors to the bacterial enzymes Rv3588 and Rv1248 was investigated by computational approaches (Figure 25 and Figure 26). A protocol consisting of classic MD and SMD simulations with molecular docking and rescoring was applied. It was proposed that the inhibitors anchor to the zinc-coordinated water molecule from the CA active site interfering with the nucleophilic attack of the zinc hydroxide on the substrate CO_2_. These compounds may be considered as interesting anti-mycobacterial lead compounds.

## 3. Conclusions

Since the first discovery of activity of simple phenol as a competitive CA inhibitor and the elucidation of its inhibition mechanism, several phenolic derivatives have been investigated in detail for their interactions with the major physiological isoforms of hCAs. Among the natural products screened so far, phenols and polyphenols remain the most investigated ones. Studies including both natural products and synthetic derivatives have allowed the identification of selective inhibitors and demonstrated the enormous potential of this class of compounds. Due to their structural variety, these products are characterized by different selectivity profiles compared to the primary sulfonamides. The latter are the classical CA inhibitors with long use in clinical practice, which nevertheless suffer from pharmacological side effects. In the search for alternative isoform-selective inhibitors phenols and polyphenols are among the most promising constituents. The different mode of mechanism, which in many cases is unknown, creates new opportunities for drug design and development. However, given the immense variety of chemotypes of natural phenolics, there is still much to be explored. Quite recently they emerged as important probes against pathogen CAs with exceptional selectivity, opening a new way for the development of novel anti-infective agents. Further studies including in vitro and in vivo tests and disease models are necessary to confirm these results.

## Figures and Tables

**Table 1:** Inhibition data of compounds **9**–**16** [45].

Compound	IC_50_ (µg/mL) ^a^	
	hCA I	hCA II
Benzoic acid (**9**)	1.31	1.12
*m*-hydroxybenzoic acid (**10**)	1.33	1.00
Propylparaben (**11**)	0.84	0.82
Protocatechuic acid (**12**)	1.31	1.15
Vanillic acid (**13**)	0.52	0.36
*o*-coumaric acid (**14**)	0.93	0.22
*trans*-cinnamic (**15**)	0.11	0.09
Chlorogenic acid (**16**)	0.98	0.88

^a^ From three different assays, errors ± 5%–10% of the reported value.

**Table 2:** Inhibition constants of certain phenolic compounds derived from guaiacol and catechol against four human carbonic anhydrase isoenzymes (hCA I, II, IX and XII) using an esterase bioassay [49].

Compound	K_I_ (μM) ^a^			
	hCA I	hCA II	hCA IX	hCA XII
Guaiacol (**17**)	7.50	5.63	9.98	9.83
4-Methylguaiacol (**18**)	9.15	7.74	9.89	8.55
4-Propylguaiacol (**19**)	10.34	8.51	9.01	9.12
Eugenol (**20**)	8.32	7.27	9.62	8.89
Isoeugenol (**21**)	10.29	6.73	9.32	9.13
Vanillin (**22**)	11.37	7.15	9.81	8.39
Syringaldehyde (**23**)	10.92	6.68	9.11	7.70
Catechol (**24**)	247.50	5.51	373.23	515.98
3-Methylcatechol (**25**)	5.95	4.69	9.55	7.22
4-Methylcatechol (**26**)	6.16	2.76	8.11	6.58
3-Methoxycatechol (**27**)	6.32	2.20	7.83	7.53

^a^ From three different assays, errors ± 5%–10% of the reported value.

**Table 3:** Inhibition of mammalian α-CA with the *C*-cinnamoyl glycosides **28**–**35** and phenol **1**.

No.	K_I_ (μM) ^a^											
	hCA I	hCA II	hCA III	hCA IV	hCA VA	hCA VB	hCA VI	hCA VII	hCA IX	hCA XII	hCA XIII	hCA XIV
**28**	8.5	7.0	>100	5.6	9.8	6.0	8.8	9.5	5.2	6.7	5.1	5.9
**29**	5.7	3.9	>100	4.9	8.4	4.0	6.2	6.3	5.9	6.2	4.9	5.6
**30**	5.1	7.1	>100	7.8	9.5	6.9	7.7	7.1	3.3	3.9	7.2	4.6
**31**	9.3	5.5	>100	6.7	9.3	5.2	7.9	5.8	2.9	4.2	6.7	2.3
**32**	6.8	7.8	>100	8.3	7.4	>100	>100	4.9	4.5	7.4	8.8	4.3
**33**	3.7	8.8	>100	7.1	4.4	>100	>100	8.5	8.2	8.7	8.6	7.1
**34**	3.6	3.1	>100	9.2	3.4	>100	8.1	9.0	9.2	8.4	8.6	>100
**35**	5.5	6.8	8.4	8.4	8.0	>100	>100	9.3	8.2	6.8	>100	>100
**1**	10.2	5.5	2.7	9.5	218	543	208	710	8.8	9.2	697	11.5

^a^ From three different assays, errors ± 5%–10% of the reported value, CO_2_ hydrase, stopped-flow assay.

**Table 4:** Structures and hCA inhibition data of dodoneine (**36**) and its analogues **37**–**41** by a stopped flow CO_2_ hydrase assay [53].

No.	K_I_ (μM) ^a^											
	hCA I	hCA II	hCA III	hCA IV	hCA VA	hCA VB	hCA VI	hCA VII	hCA IX	hCA XII	hCA XIII	hCA XIV ^b^
**36**	5.48	_ ^c^	10.35	9.61	_ ^c^	_ ^c^	_ ^c^	_ ^c^	_ ^c^	_ ^c^	9.27	9.34
**37**	0.38	_ ^c^	_^c^	4.12	21.6	13.7	_ ^c^	_ ^c^	32.6	24.5	8.13	7.24
**38**	0.76	21.8		13.7	8.55	6.32			15.7	10.8	7.89	12.5
**39**	0.13	36.9		5.36	7.13	1.36	_ ^c^	24.9	3.57	1.48	0.96	2.44
**40**	_ ^c^	_ ^c^	10.80	_ ^c^	_ ^c^	_ ^c^	_ ^c^	_ ^c^	_ ^c^	_ ^c^	0.91	_ ^c^
**41**	_ ^c^	_ ^c^	5.13	_ ^c^	_ ^c^	_ ^c^	_ ^c^	_ ^c^	_ ^c^	_ ^c^	0.34	_ ^c^

^a^ From three different assays, errors ± 5%–10% of the reported value. ^b^ mCA; ^c^ Not active > 100.

**Table 5:** Inhibition data of human CA isoforms hCA I, II, IV, VII and XII with salvianolic acids **43**–**45** reported here and the standard sulfonamide inhibitor acetazolamide (**AAZ**) by a stopped flow CO_2_ hydrase assay [58].

No.	K_I_ (nM) ^a^				
	hCA I	hCA II	hCA IV	hCA VII	hCA XII
Salvianolic Acid A (**43**)	>10,000	9594.4	66.6	71.4	39.8
Lithospermic Acid (**44**)	>10,000	>10,000	101.2	268.3	4.8
Salvianolic Acid B (**45**)	>10,000	>10,000	65.6	35.5	453.6
**AAZ**	250	12	74	2.5	5.7

^a^ From three different assays, errors ± 5%–10% of the reported value.

**Table 6:** Inhibition of hCA isozymes I, II and IV and bCA III with compounds **60**–**64**, **EZA**, **ZNA** and **AZA** by the esterase activity.

No.	IC50 Value (µM) ^a^			
	hCA I	hCA II	bCA II	hCA IV
Apigenin (**60**)	4.1	2.7	11.6	9.1
Luteolin (**61**)	2.2	0.74	5.4	4.4
Quercetin (**62**)	3.6	2.4	9.1	13.8
Morin (**63**)	12.8	4.4	21.3	15.7
Catechin (**64**)	6.8	6.2 ^b^	2.2	5.6
Phenol	17.3	7.4	4.6	14.3
Catechol	4014	11.4	6.5	11.9
Resorcinol	828	9.4	219	634
**EZA**	3.7	0.32	9.4	0.84
**ZNA**	14.8	1.1	8.5	38.4
**AZA**	36.2	0.37	263	0.58

^a^ From three different assays, errors ± 5%–10% of the reported value. ^b^ Values from Ref. [66].

**Table 7:** hCA isoforms I and II inhibition with compounds **60**, **64**–**70** by a stopped-flow, CO_2_ hydration assay [46].

No.	IC_50_ Value (µg/mL) ^a^	
	hCA I	hCA II
Apigenin (**60**)	1.22	1.14
Catechin (**64**)	1.24	0.87
Kaempferol (**65**)	0.83	0.76
Fisetin (**66**)	0.72	0.55
Rhamnetin (**67**)	0.95	0.86
Isorhamnetin (**68**)	0.52	0.36
Rutin (**69**)	1.44	0.52
Epicatechin (**70**)	1.47	1.02

^a^ From three different assays, errors ± 5%–10% of the reported value.

**Table 8:** K_I_ values (μM) for compounds **60**, **62**, **64**, **69**–**71** and **AZA** of some human (h) and bovine (b) α-carbonic anhydrase isoforms (by an esterase assay) [67].

No.	K_I_ (nM) ^a^				
	hCA I	hCA II	bCA III	hCA IV	hCA VI
Apigenin (**60**)	0.97	0.113	2.21	1.12	1.49
Quercetin (**62**)	2.68 ^b^	2.54 ^b^	3.73	7.89 ^b^	6.17 ^b^
Catechin (**64**)	1.73	0.83	4.77	3.76	7.82
Rutin (**69**)	2.42 ^b^	1.84 ^b^	9.71	4.09 ^b^	4.91 ^b^
Epicatechin (**70**)	2.32	1.24	8.93	3.98	4.36
Silymarin (**71**)	1.49 ^c^	2.51 ^c^	5.68	8.96 ^c^	9.70 ^c^
**AZA**	36.2	0.37	263	0.578	0.34

^a^ From three different assays, errors ± 5%–10% of the reported value. ^b^ Values from Ref. [68]; ^c^ Values form Ref. [66].

**Table 9:** Inhibition constant K_I_ values (μM) of human carbonic anhydrase I, II, VA, IX, and XII isoforms for compounds **60**, **62**, **65**, **68**, **72**–**77** and acetazolamide (**AZA**).

No.	K_I_ (nM) ^a^				
	hCA I	hCA II	hCA VA	hCA IX	hCA XII
Apigenin (**60**)	4.10	2.70 ^b^	0.30	0.46	1.00
Quercetin (**62**)	2.95	0.41	2.82	2.52	0.38
Kaempferol (**65**)	3.13	0.31	0.15	3.57	2.18
Isorhamnetin (**67**)	>10	0.42	1.92	0.44	0.29
Diosmetin (**72**)	>10	>10	2.10	3.39	0.24
Eriocitrin (**73**)	3.66	0.34	4.24	0.43	0.19
Hesperidin (**74**)	3.60 ^b^	2.40 ^b^	2.65	2.22	0.45
Naringin (**75**)	>10	0.41	4.08	3.59	0.17
Taxifolin (**76**)	2.53	0.26	3.93	0.45	0.11
Quercetin-3-*O*-glucoside (**77**)	>10	0.44	>10	2.79	0.22
**AZA**	0.10	0.008	0.38	0.041	0.038

^a^ From three different assays, errors ± 5%–10% of the reported value, CO_2_ hydrase, stopped-flow assay. ^b^ Values from Ref. [65].

**Table 10:** Inhibition data of human CA isoforms hCA I, II, IV, VII and XII with flavonoids **59**, **63**, **66**, **74**, **75**, **76**–**88** reported here and the standard sulfonamide inhibitor acetazolamide (**AAZ**) by a stopped flow CO_2_ hydrase assay.

Entry	Name	K_I_ (nM) ^a^				
		hCA I	hCA II	hCA IV	hCA VII	hCA XII
**79**	Chrysin	>10,000	>10,000	537.75	171.41	34.7
**61**	Luteolin	>10,000	3691.6	293.0 ^b^	4.7	60.3
**80**	Galangin	>10,000	>10,000	568.3	24.5	41.8
**65**	Kaempferol	>10,000	9525.7	>10,000	25	145.9
**67**	Isorhamnetin	>10,000	>10,000	212.9	4.4	54.9
**81**	Naringenin	>10,000	>10,000	79.5	4.3	44.3
**82**	Eriodictyol	>10,000	>10,000	72.8	4.3	31.1
**83**	Hesperitin	>10,000	>10,000	102.1	3.3	454.1
**76**	Taxifolin	>10,000	>10,000	9098.2	493.1	32.5
**84**	Daidzein	>10,000	>10,000	718.7	4.2	56.4
**85**	Biochanin A	>10,000	>10000	7078.5	371.5	52.5
**86**	Puerarin	>10,000	>10,000	>10,000	452.6	515.0
**77**	Quercetin-3-*O*-glucoside	>10,000	6211.2	75.7	3.8	52.1
**87**	Quercetin-3-*O*-rhamnoside	>10,000	6367.7	67.3	3.9	43.5
**88**	Kaempferol-3-*O*-glucoside	>10,000	168.0	5591.2	423.7	4.9
**89**	Tiliroside	>10,000	>10,000	5468.7	4.6	134.3
**90**	Kaempferol-3-*O*-(2″,6″-di-*E*-p-coumaroyl)-β-glucopyranoside	>10,000	8790.2	344.2	4.7	408.9
**91**	Kaempferol-3-*O*-(3″,4″-diacetyl-2″,6″-di-*E-*p-coumaroyl)-β-glucopyranoside	>10,000	6863.3	62.2	26.6	399.7
	**AAZ**	250	12	74	2.5	5.7

^a^ Mean from three different assays, errors were in the range of ± 5%–10% of the reported values. ^b^ From Ref. [65].

**Table 11:** K_I_ values of the compounds **92**–**103** (CA esterase activity).

Compound	K_I_ (nM) ^a^	
	hCA I	hCA II
**92**	274.19	645.39
**93**	112.34	154.45
**94**	325.12	418.06
**95**	165.35	227.56
**96**	308.88	307.52
**97**	111.22	193.19
**98**	177.91	446.62
**99**	188.56	208.53
**100**	323.30	182.31
**101**	72.90	166.54
**102**	179.11	268.87
**103**	103.55	319.13
**AZA**	250.00	12.00

^a^ Mean from at least three determinations. Errors in the range of ~3% of the reported value.

**Table 12:** Enzyme inhibition of pathogenic *M. tuberculosis* β-CA isozymes Rv3273 and Rv1284, *C. albicans* isozyme Nce103, *C. neoformans* isozyme Can2 and human α-CA isozymes I and II, with the NP-Based Library (**104**–**124**), known CA Inhibitors (SA, AZA, ZNS, and TPM) and phenol.

Compound	K_I_ (µM) ^a^					
	Rv3273	Rv1284	Nce103	Can2	CA I	CA II
**104**	12.1	0.85	1.10	1.08	430	8.711.4
**105**	11.4	10.8	1.02	0.90	309	10.3
**106**	9.12	0.85	0.91	0.84	309	11.2
**107**	10.8	10.3	1.08	1.12	265	8.6
**108**	11.2	10.5	1.00	0.85	237	131
**109a**	11.3	0.84	1.03	1.15	239	8.3
**109b**	10.9	0.71	1.06	1.11	231	8.0
**110**	0.91	11.8	0.92	0.89	10.5	9.6
**111**	0.92	0.91	0.90	0.95	355	13.1
**112**	11.4	10.5	1.06	1.12	201	8.4
**113**	10.9	0.99	1.01	1.08	374	9.2
**114**	8.92	0.82	0.73	0.77	368	11.7
**115**	0.89	0.80	0.70	0.95	354	12.1
**116**	9.10	0.85	0.62	0.81	307	230
**117**	0.98	12.2	0.78	0.72	10.5	11.4
**118**	0.97	0.80	0.93	0.81	9.6	9.8
**119**	0.91	1.27	0.72	0.94	11.2	10.8
**120**	0.90	1.78	0.75	0.86	11.9	11.5
**121**	0.85	1.16	0.79	0.74	0.70	0.018
**122**	1.14	11.0	0.99	0.95	158	10.4
**123**	10.2	12.3	0.96	0.91	11.4	10.8
**124**	10.4	11.6	0.81	0.73	10.7	9.4
**SA**	7.11	9.84	7.63	0.77	25.0	0.24
**AZA**	0.10	0.48	0.13	0.01	0.25	0.012
**TPM**	3.02	0.61	1.11	0.37	0.25	0.010
**ZNS**	0.21	286.8	0.94	0.97	0.056	0.035
Phenol	79.0	64.0	17.3	25.9	10.1	5.5

^a^ Errors in the range of ±5% of the reported value, from three determinations.

**Table 13:** Inhibition of human CA isozymes I and II and Mtb CAs Rv1248, Rv3273 and with phenolic acids **6**–**8** and esters **122**–**129** by a stopped flow CO_2_ hydrase assay.

Compound	K_I_ (µM) ^a,b^				
	hCA I	hCA II	Rv1248	Rv3273	Rv3588
**6**	1.07	0.98	6.05	2.69	4.33
**7**	2.38	1.61	5.92	6.70	5.36
**8**	2.89	2.40	7.13	2.40	5.64
**125**	5.23	>50	5.32	5.10	7.13
**126**	9.62	>50	3.95	6.83	8.03
**127**	>50	>50	4.51	3.19	7.05
**128**	>50	>50	5.67	3.20	7.26
**129**	>50	>50	4.69	2.32	5.40
**130**	>50	>50	3.78	1.84	5.04
**131**	3.87	>50	4.67	1.87	5.13
**132**	3.66	>50	5.74	2.30	6.09

^a^ Errors in the range of ±5% of the reported data from three different assays. ^b^ h = human.
